# miR-145 sensitizes breast cancer to doxorubicin by targeting multidrug resistance-associated protein-1

**DOI:** 10.18632/oncotarget.10845

**Published:** 2016-07-26

**Authors:** Man Gao, Lingling Miao, Mingxia Liu, Chenggang Li, Cunzhi Yu, Hong Yan, Yongxiang Yin, Yizheng Wang, Xinming Qi, Jin Ren

**Affiliations:** ^1^ Center for Drug Safety Evaluation and Research, Shanghai Institute of Materia Medica, CAS., Shanghai, P.R. China; ^2^ Department of Pathology, Wuxi Maternity and Children Health Hospital Affiliated Nanjing Medical University, Wuxi, China; ^3^ The Brain Science Center, Beijing Institute of Basic Medical Sciences, Beijing, China

**Keywords:** breast cancer, MRP1, miRNA, chemoresistance, doxorubicin

## Abstract

Multidrug resistance-associated protein 1 (MRP1) is an important efflux transporter and overexpression of MRP1 usually leads to chemoresistance in breast cancer. Here, we found MRP1 overexpressed in human breast cancer tissues and breast cancer cell lines (compared with normal breast tissues and cell line, respectively). And MRP1 level increased in doxorubicin resistant MCF-7 cells compared with parental MCF-7 cells. Increasing evidences suggest microRNAs (miRNAs) influence chemotherapy response. We found miR-145 level decreased in human breast cancer tissues, breast cancer cell lines and doxorubicin resistant MCF-7 cells, and inversely correlated with MRP1 expression level. In the process of constructing MCF-7 doxorubicin resistant cell line, escalating doxorubicin markedly decreased miR-145 level, following by increased MRP1 level. Further study showed, miR-145 suppressed MRP1 expression by directly targeting MRP1 3′-untranslated regions. Overexpression of miR-145 sensitized breast cancer cells to doxorubicin *in vitro* and enhanced to doxorubicin chemotherapy *in vivo* through inducing intracellular doxorubicin accumulation via inhibiting MRP1. Taken together, our study revealed miR-145 sensitizes breast cancer to doxorubicin by targeting MRP1 and indicated the potential application in developing MRP1 inhibitor.

## INTRODUCTION

Breast cancer is the second leading cause of mortality among all types of cancer in women [1–3]. Surgical treatment and chemotherapy are the main treatment strategies for breast cancer [4, 5]. However, many patients receiving chemotherapy exhibit a poor initial response or gradually develop resistance to chemotherapy, which is defined as multidrug resistance (MDR) and perhaps the greatest obstacle to the treatment of breast cancer. According to our current understanding, the mechanisms underlying drug resistance can be broadly divided into two categories. First, the actual drug targets undergo functional or structural alterations that decrease the effect of the drugs; alternatively, a series of alterations to the components of signal transduction pathways or to key regulators of cell death execution suppress the effect of the drugs [6]. Second, the normal transport between tumor cells and chemotherapeutic agents is destroyed; this mechanism refers to the adaptions that decrease drug uptake and increase drug export, ultimately reduce intracellular concentration of the chemotherapeutic agent [7, 8]. Drug transporters play an important role in this type of drug resistance, and MDR-associated protein 1 (MRP1) is one of the most thoroughly studied transporters related to drug resistance [9].

The 190-kDa protein MRP1, which is encoded by ABCC1 gene and belongs to the human ATP-binding cassette (ABC) superfamily, is an important efflux transporter. A broad range of anti-cancer drugs are extruded by MRP1 [10, 11]. MRP1 overexpression often accompanies drug resistance emerging [12].

MicroRNAs (miRNAs) are a class of endogenous, small non-coding RNAs of 20-25 nucleotides in length that can regulate gene expression [13]. More than 60% of human genes are regulated by miRNAs, which can block the translation or induce the degradation of target messenger RNAs via sequence-specific hybridization to the 3′-untranslated region (UTR) of the target [14]. Previous studies suggest multiple miRNAs are dysregulated when drug resistance developed, which indicates that miRNAs might play an important role in drug resistance [15–17]. It has been reported that miR-326 and miR-133a reduce adriamycin resistance of human hepatoma HepG2 cells through downregulating ABCC1 expression [18]; miR-1291 could modulate cellular drug disposition through direct targeting ABCC1 in PANC-1 cells [19]. These studies suggest that miRNAs may influence chemotherapy response by inhibiting MRP1 in liver and pancreatic cancer cells.

We analyzed breast cancer dataset from TCGA and found that the levels of miR-326, miR-133a and miR-1291 were all very low, previously reported miRNAs targeting MRP1 in other cancers [18, 19] and their expression levels showed no markedly significant difference in tumors compared to their normal adjacent tissues in breast cancer (Supplementary Figure S1).

In this study, firstly we predicted miRNAs potentially binding to the 3′UTR of ABCC1 using bioinformatics approach, then, screened the identified miRNAs for their ability to decrease MRP1 protein expression and found miR-145 is a strong candidate significantly reducing MPR1 expression in breast cancer.

Here, we investigated the role of miR-145 in the drug resistance of breast cancer. We found that miR-145 was significantly downregulated in breast cancer clinical samples and inversely correlated with MRP1 expression level in both breast cancer cell lines and clinical breast cancer tissues. We further demonstrated that miR-145 negatively regulated MRP1 expression by directly targeting MRP1 3′UTR in MCF-7 cells. For drug resistance, *in vitro*, miR-145increased sensitivity to ADR by inducing intracellular doxorubicin accumulation via inhibiting MRP1 expression. *In vivo*, forced expression of miR-145 increased sensitivity to doxorubicin in MDA-MB-231 cell-based xenograft of nude mice. Our results proved that miR-145 sensitized breast cancer to doxorubicin chemotherapy and provide a new strategy for the development of MRP1 modulators.

## RESULTS

### Identification of miRNAs downregulating MRP1

**Figure 1:**
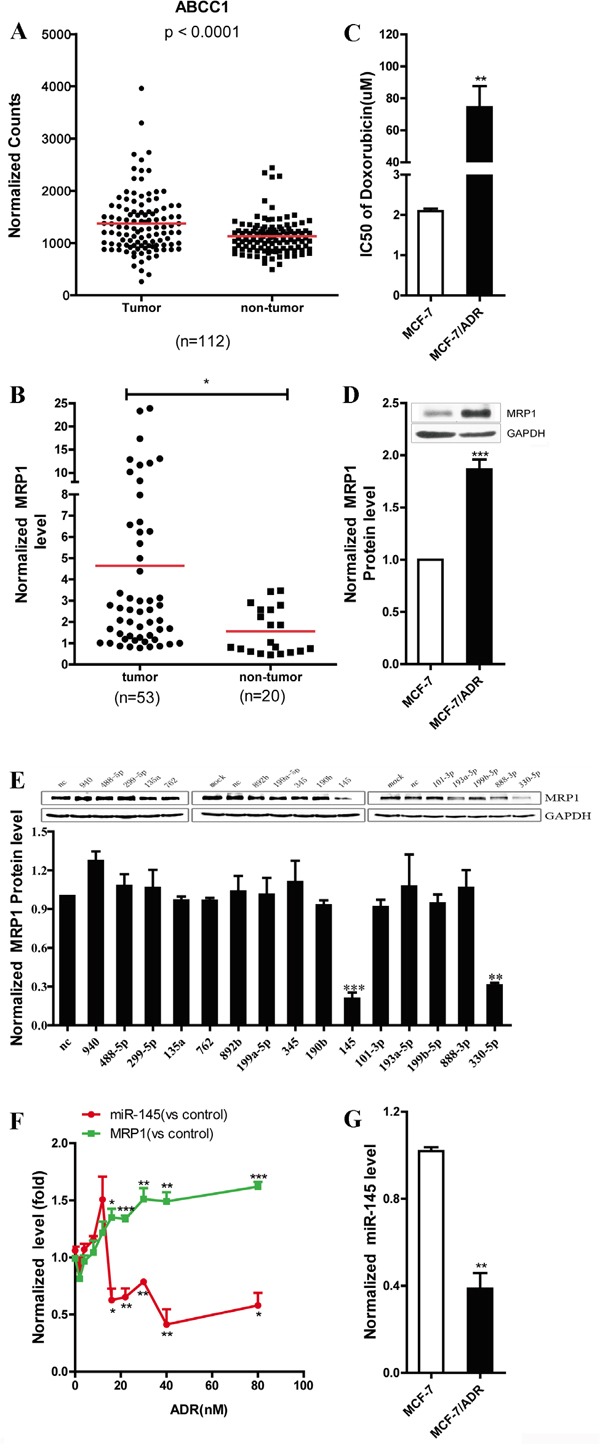
Screening for the miRNAs that decreased MRP1 expression in breast cancer **A.** Statistical analysis of the data for the MRP1 expression in breast cancer from the TCGA dataset. The MRP1 mRNA expression level was compared between tumor samples and matched normal samples (n = 112). **B.** Quantification of the MRP1 level by real-time PCR in clinical breast cancer tissue (n=53) and normal breast tissue (n=20). GAPDH was used as an internal control. **C.** Identify the sensitivity of MCF-7 and MCF-7/ADR to doxorubicin by MTT assay. **D.** Comparison of the MRP1 protein expression level in MCF-7 and MCF-7/ADR by western blot. GAPDH was used as an internal control. **E.** Screening for the miRNAs that decreased MRP1 expression in breast cancer by western blot. **F.** Determination of MRP1 and miR-145 expression level in the process of inducing doxorubicin resistance in MCF-7 cells by real time PCR. Parental MCF-7 was used as control. GAPDH and U6 snRNA was used as internal control. **G.** Comparison of the miR-145 expression level in MCF-7 and MCF-7/ADR by real-time PCR, U6 snRNA was used as an internal control.

Analysis of MRP1 expression data in breast cancer from TCGA database revealed that MRP1 expression level was significantly higher in tumor than in the matched normal breast (Figure 1A). A total of 53 breast cancer tissues and 20 non-tumor breast tissues were collected from hospital, and mRNA expression level of MRP1 was detected via real-time PCR. We found that MRP1 mRNA expression level was markedly higher in breast cancer tissues than in non-tumor breast tissues (Figure 1B), and this result was consistent with the data obtained from the TCGA database (Figure 1A). Further, MRP1 was overexpressed in doxorubicin resistant MCF-7 cells (MCF-7/ADR) compared with parental MCF-7 cells (Figure 1C and 1D). Using TargetScan 5.1 and MicroCosm, we predicted that 15 miRNAs potentially binding to the 3′UTR of human MRP1. To identify the miRNAs that negatively regulated human MRP1 in breast cancer, 50 nM mimics of individual miRNAs were transfected into MCF-7 cells. Then, western blot was used to detect the expression level of MRP1. The results indicated that miR-330-5p and miR-145 markedly inhibited MRP1 expression (Figure 1E). In this study, we limited our investigation to miR-145. We examined miR-145 and MRP1 expression level in the process of inducing MCF-7 doxorubicin resistance and the data showed: a) miR-145 expression level increased a little firstly and then decreased markedly; b) this decrease was followed by a time-dependent increase of MRP1 mRNA level, a strong indication of miR-145 action in MRP1-mediated doxorubicin resistance in MCF-7 cells (Figure 1F). Besides, miR-145 was downregulated in MCF-7/ADR cells (Figure 1G).

### miR-145 level inversely correlates with MRP1 mRNA level in breast cancer

**Figure 2:**
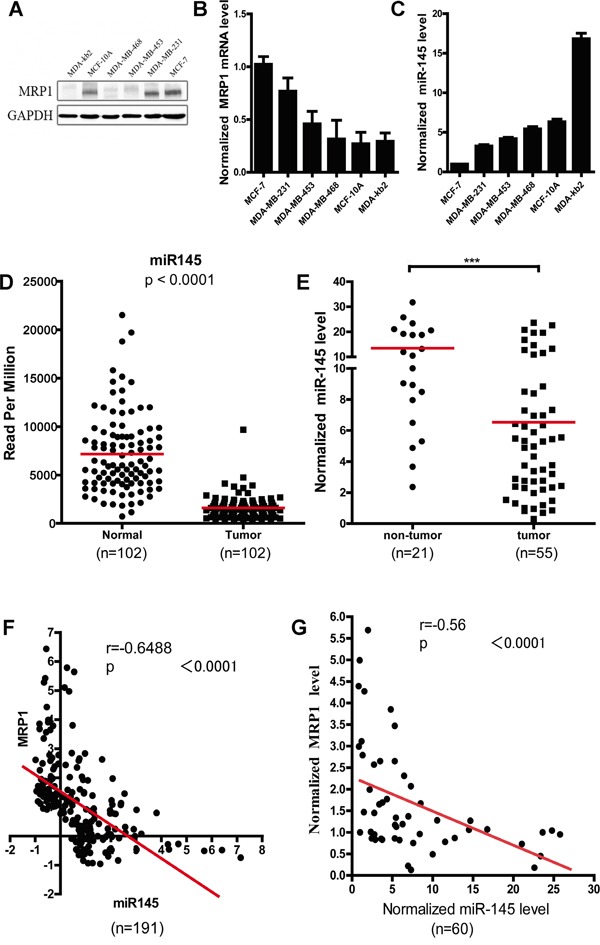
Inverse correlation between the miR-145 and MRP1 expression level in breast cancer **A.** and **B.** Examination of the MRP1 protein and mRNA expression level in different breast cancer cell lines. GAPDH was used as an internal control. **C.** Detection of the miR-145 expression level in different breast cancer cell lines. U6 snRNA was used as an internal control, and MCF-7 was used as the control cell line. **D.** Statistical analysis of the data for miRNA expression in breast cancer from the TCGA dataset. The miR-145 expression level was compared between tumor samples and matched normal samples (n = 102). **E.** Quantification of the miR-145 levels via real-time PCR in clinical breast cancer tissues (n=55) and normal breast tissues (n=21). U6 snRNA was used as an internal control. **F.** and **G.** Spearman's correlation analysis of the correlation between the miR-145 and MRP1 mRNA expression level in breast cancer. (F) Data obtained from the TCGA database (n=191); (G) data obtained via real-time PCR.

To determine whether there was an inverse correlation between MRP1 and miR-145 expression level in breast cancer, we tested the MRP1 and miR-145 expression level in two normal human mammary epithelial cell lines (MCF-10A and MDA-kb2) and four breast cancer lines (MCF-7, MDA-MB-231, MDA-MB-453 and MDA-MB-468); MCF-7 was used as a control cell line. We found that the breast cancer cell lines displayed lower endogenous miR-145 expression level than the normal human mammary epithelial cell lines (Figure 2C). Further, our results showed that miR-145 level inversely correlated with the MRP1 mRNA level in the breast cancer and normal human mammary epithelial cell lines examined (correlation coefficient R= -0.9771; P=0.0042) (Supplementary Figure S2). MCF-10A and MDA-kb2 cells, which displayed high miR-145 expression level, exhibited relatively low MRP1 expression, whereas MCF-7, MDA-MB-231, MDA-MB-453 and MDA-MB-468 cells, which displayed low miR-145 level, exhibited relatively high MRP1 expression level (Figure 2B and 2C).

We further analyzed the data of miR-145 expression level in breast cancer from TCGA database and found miR-145 expression level in tumor samples were significantly decreased compared to that in the matched normal samples (Figure 2D). In our collection (55 breast cancer tissues and 21 breast non-tumor tissues), the miR-145 expression level was also significantly lower in the breast cancer tissues than in the non-tumor breast tissues (Figure 2E). And, we analyzed miR-145 expression level in different molecular subtypes of breast cancer, but no significant difference in miR-145 expression between different molecular subtypes was observed (Table 1).

**Table 1:** miR-145 level in different molecular subtypes of breast cancer

	miR-145 (per million)
HER2-enriched (n=34)	1442
Luminal A (n=400)	1429
Luminal B (n=106)	1555
TNBC (n=112)	1484

Spearman's correlation analysis of the data of miR-145 and MRP1 expression level in breast cancer from TCGA database and our collection showed an inverse correlation between miR-145 level and MRP1 expression level (correlation coefficient R= -0.6488; P<0.0001 in TCGA; R= -0.58; P<0.0001 in our collection) (Figure 2F and 2G). Moreover, this inverse correlation was observed in both luminal A and luminal B breast cancer (Table 2).

**Table 2:** correlation between the miR-145 and MRP1 level in different molecular subtypes of breast cancer

	miR-145	MRP1	n	Spearman Test	
				*p*-value	*r-*value
Luminal A	0.222	0.365	235	<0.0001	−0.2778
Luminal B	0.097	0.598	72	0.0016	−0.3652
TNBC	0.276	1.956	43	0.2902	−0.1651

### MPR1 is a direct target of miR-145

**Figure 3:**
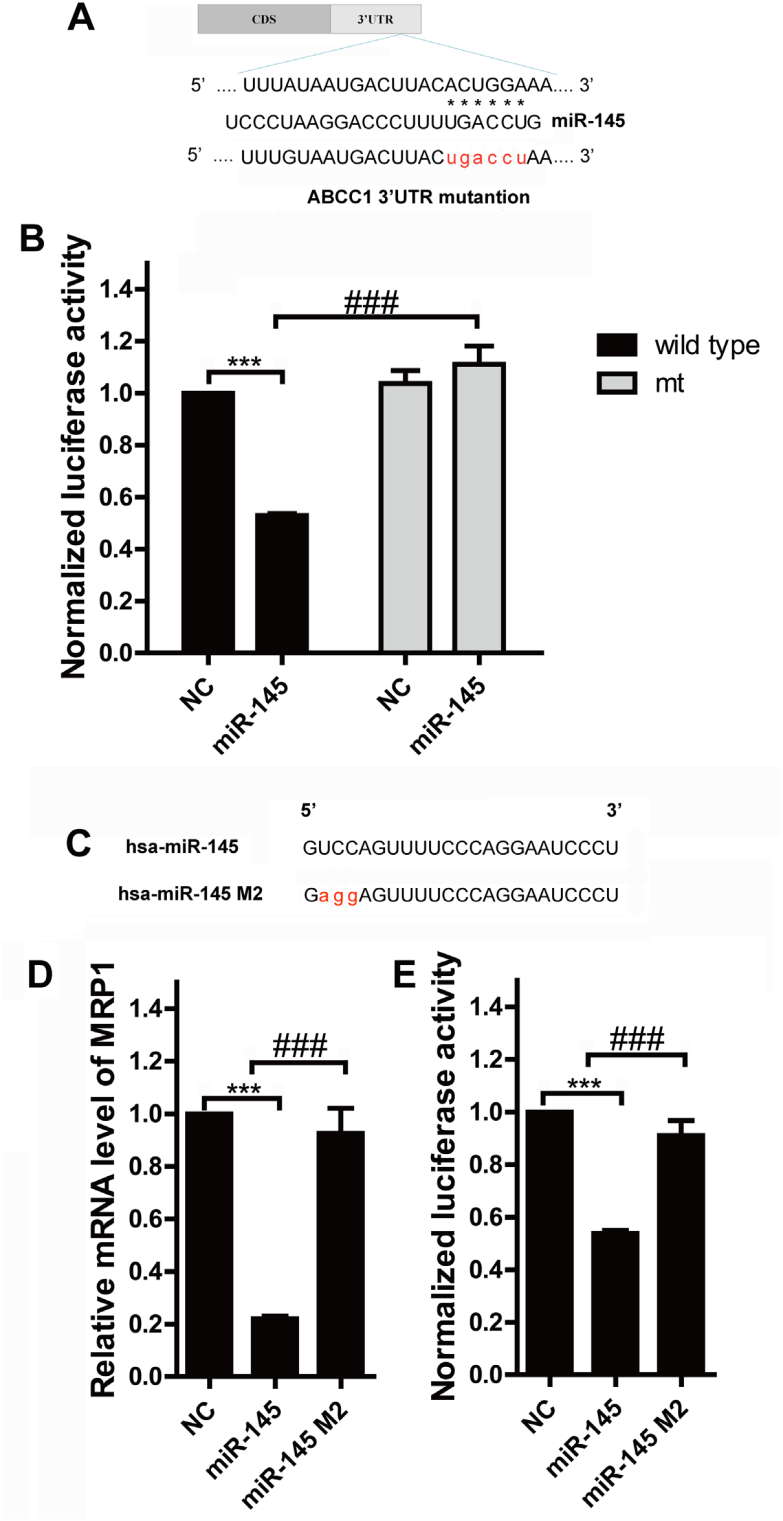
MRP1 is a direct target of miR-145 in breast cancer **A.** miR-145 and its putative binding sequence in the 3′UTR of ABCC1. A mutant miR-145 binding site was generated in the complementary site for the seed region of miR-145 (wt, wild type; mt, mutant). **B.** Luciferase assay for the direct targeting of 3′UTR of ABCC1 by miR-145. The wild-type or miR-145 binding sequence mutated luciferase reporter plasmids were co-transfected with miR-145 mimics or negative control oligonucleotide and then luciferase activity was analyzed. Each bar represents the relative luciferase activity. **C.** Synthesis of the mutant of miR-145 in which a sequence of the conserved MRP1-binding base was specifically mutated; this sequence was labeled as miR-145 M2. **D.** and **E.** Identify the effect of miR-145 M2 on MRP1. miR-145 mimics, miR-145 M2 oligonucleotide or negative control oligonucleotide was transfected into MCF-7, or co-transfected with wild-type luciferase reporter plasmid into MCF-7, MRP1 mRNA expression level and luciferase was examined.

The 3′UTR of human ABCC1 was amplified, and the resulting PCR fragments were cloned into the psiCHECK-2 vector (Figure 3A, labeled as wild type ABCC1 3′UTR). In a parallel experiment, the predicted targeting region for miR-145 binding (nt 1728-1734, ACUGGA), was mutated (Figure 3A, labeled as mt ABCC1 3′-UTR). Co-transfecting the wild type ABCC1 3′UTR plasmid and miR-145 mimics into MCF-7 cells significantly decreased luciferase activity compared with the control (Figure 3B, P<0.0001). And luciferase activity was restored when mt ABCC1 3′UTR plasmid and miR-145 mimics were co-transfected into MCF-7 cells (Figure 3B, P<0.0001). These results suggested that the conserved 1728-1734 nt ACUGGA region within the ABCC1 3′UTR is responsible for the binding between miR-145 and MRP1 and MRP1 is a direct target of miR-145. To further demonstrated that miR-145 targeted the MRP1 3′UTR, we synthesized a miR-145 mutant in which a portion of its conserved targeting region (UCC) was specifically mutated (Figure 3C, labeled as has-miR-145 M2). MCF-7 cells were transfected with miR-145 or miR-145 M2, respectively. miR-145 decreased MRP1 expression, but miR-145 M2 did not show this suppressive effect on MRP1 expression (Figure 3D). Similarly, co-transfecting miR-145 M2 and wild type ABCC1 plasmid eliminated the function of co-transfecting miR-145 and wild type ABCC1 plasmid on luciferase activity (Figure 3E). Thus, miR-145 suppresses MRP1 via binding to MRP1 3′UTR.

### miR-145 sensitizes breast cancer cells to doxorubicin through inhibiting MRP1

**Figure 4:**
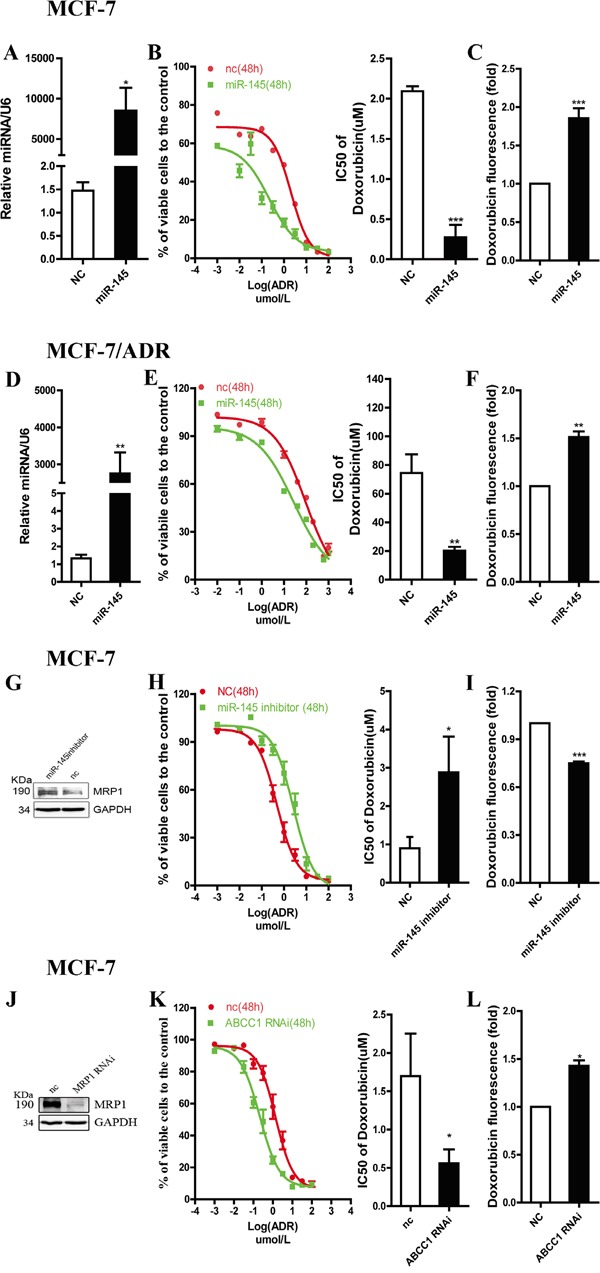
miR-145 regulates drug sensitivity of breast cancer cell lines to ADR through targeting MRP1 **A.** and **D.** The expression of miR-145 was examined by qRT-PCR in cells transfected with the mimics. U6 snRNA was used as an internal control. **G.** and **J.** MRP1 expression level was detected by western blot in cells transfected with miR-145 inhibitors or ABCC1 siRNA, GAPDH was used as an internal control. **B.** **E.** **H.** and **K.** ADR sensitivity of MCF-7 and MCF-7/ADR were tested by MTT assay. **C.** **F.** **I.** and **L.** Intracellular doxorubicin accumulation was measured by flow cytometry. Relative drug accumulation indicated by geometry mean of doxorubicin fluorescence.

Doxorubicin (adriamycin, ADR), a substrate of MRP1, is a classical agent used for breast cancer chemotherapy [11, 20]. Thus, we chose ADR to investigate the role of miR-145 in MRP1-related drug resistance. miR-145 mimics (50 nM) were transfected into MCF-7 or MCF-7/ADR cells to increasemiR-145 level (Figure 4A and 4D). As suggested by a decline in the IC_50_ values (Figure 4B and 4E), miR-145 overexpression markedly sensitized cells to ADR. The major mechanism by which MRP1 participates in drug resistance is drug efflux, which reduces the intracellular drug concentration. To determine whether miR-145 sensitized cells to ADR via MRP1, we detected the intracellular ADR concentration in MCF-7 or MCF-7/ADR cells transfected with miR-145 mimics. The results showed that miR-145 increased the intracellular ADR concentration (Figure 4C and 4F). These results provide evidence that miR-145 sensitizes breast cancer cells to ADR via MRP1. To examine the effect of endogenous miR-145, antisense oligonucleotide against miR-145 (50 nM) was transfected into MCF-7 cells. As a consequence, MRP1 expression level was increased (Figure 4G), conversely, the miR-145 level was reduced; leading to an increase in the IC_50_ value of ADR (Figure 4H) and a decrease in the intracellular concentration of ADR (Figure 4I). To confirm the function of MRP1 on ADR resistance in MCF-7 cells, chemically synthesized MRP1 siRNA was transfected into MCF-7 cells and MRP1 expression level was examined via western blot (Figure 4J). Silencing MRP1 by siRNA significantly sensitized MCF-7 cells to ADR by increasing the intracellular concentration of ADR (Figure 4K and 4L). These data suggest miR-145 sensitizeed MCF-7 cells to ADR via inducing intracellular ADR accumulation by inhibiting MRP1.

### Restoration of miR-145 expression sensitizes breast cancer cells to ADR by targeting MRP1 *in vivo*

**Figure 5:**
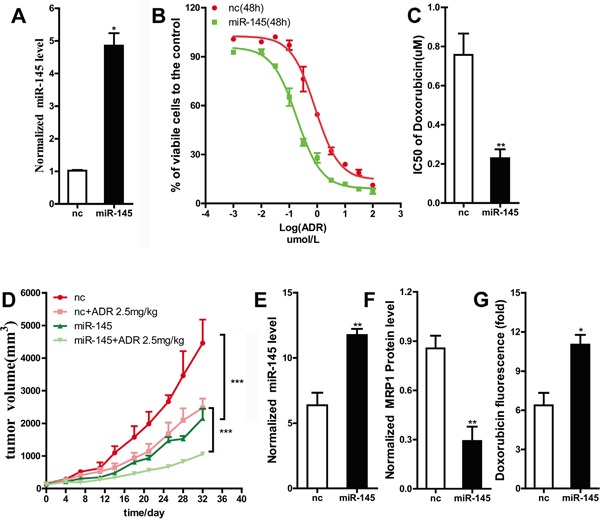
The restoration of miR-145 sensitized MDA-MB-231 cells to doxorubicin *in vivo* **A.** The expression level of miR-145 in MDA-MB-231 cells stably transfected with leti-miR-145 was tested by qRT-PCR, U6 snRNA was used as an internal control. **B.** and **C.** ADR sensitivity of MDA-MB-231 cells stably transfected with leti-miR-145 was examined by MTT assay. **D.** Tumor growth curves of orthotopic implantation models are shown. The tumor volumes were measured at the indicated time points. The tumor volumes calculated as length ×width^2^. **E.** and **F.** The expression level of miR-145 and MRP1 in group nc and group miR-145 were analyzed. **G.** The concentration of doxorubicin in group nc +ADR and miR-145 +ADR were measured.

MDA-MB-231 is one of the triple negative breast cancer cell lines, and the agent doxorubicin which is classical substrate for MRP1, is used for the treatment of triple negative breast cancer patients. Additionally, MCF-7 cell lines in our laboratory did not show any ability of forming tumor in nude mice, so MDA-MB-231 cell line was chose to validate the action of miR-145 in-vivo. In our study, lentivirus-mediated transfection was used to obtain a cell line stably overexpressing miR-145 (Figure 5A). Then, we examined the sensitivity of this stable miR-145-overexpressing cell line to ADR chemotherapy using the MTT assay. As shown by a decline in the IC_50_ value of ADR (Figure 5B and 5C), this cell line was markedly sensitized to ADR chemotherapy compared to the control cell line. To investigate whether miR-145 increases breast cancer drug sensitivity *in vivo*, MDA-MB-231 cells stably expressing a control vector or miR-145 were subcutaneously injected into the right fat pad of nude mice. The tumor volume was monitored every three days. Significant difference in the tumor volume was observed between the NC group and the NC + ADR chemotherapy group (Figure 5D), and between the miR-145 group and the miR-145 + ADR chemotherapy group (Figure 5D). In this study, the tumor volume of miR-145 group was smaller than that of NC group. This observation showed that miR-145 suppressed breast cancer growth *in vivo* (Figure 5D), consistent with previous report [21].

The markedly differences in the tumor volume between the NC + ADR chemotherapy group and the miR-145 + ADR chemotherapy group (Figure 5D) suggested that ectopic miR-145 expression can sensitize breast cancer to ADR chemotherapy. The mice were humanely sacrificed at 32 days, and their tumors were harvested. RNA and protein were extracted from the tumor tissues. The qRT-PCR and western blot data showed that the protein expression level of MRP1 was decreased due to the overexpression of miR-145; this result was consistent with the *in vitro* findings (Figure 5E, 5F). We also examined the concentration of ADR in tumor tissue. Compared to the control group, the miR-145 group displayed an increased ADR concentration (Figure 5D). This result further indicated that miR-145 sensitized breast cancer to ADR chemotherapy via increasing intracellular ADR accumulation by reducing the MRP1 expression level. This result was also consistent with our *in vitro* findings in breast cancer cell lines.

## DISCUSSION

It has been known that MRP1 is a drug resistance-associated ABC transporter in various cancers. And increasing evidences suggest miRNAs play an important role in MDR by regulating MDR-associated transporters, hence, identification of miRNAs regulating the expression of MDR-associated transporters such as MRP1 have great significance for overcoming chemoresistance. Here, we demonstrated that miR-145 sensitized breast cancer to doxorubicin both *in vitro* and *in vivo* by suppressing MRP1 expression.

MRP1 is a multidrug efflux pump that plays an important role in the uptake and distribution of anti-cancer agents [22]. Elevated expression of MRP1 is frequently observed in MDR cancer cases and is closely correlated to the outcome of chemotherapy [23–25]. In our study, we analyzed the data of MRP1 expression level in breast cancer from TCGA database and from clinical samples. MRP1 expression level was significantly higher in breast cancer tissues than in non-tumor breast tissues (Figure 1A and 1B). Moreover, analysis of the data from TCGA database revealed that MRP1 expression level in breast cancer samples was higher than that of most other ABC transporters, such as ABCB1 and ABCG2, which are reported to be associated with drug resistance (Supplementary Figure S3). This finding also indicated that MRP1 mayhave more important role in drug resistance of breast cancer. MDR modulators are a group of compounds which act as chemotherapy inhibitors or sensitizers [26, 27]. However, there is still no this kind inhibitors suitable for clinical treatment. The development of MDR modulators is a challenging task for anti-cancer chemotherapy.

Recent studies have shown that miRNAs may play an important role in MRP1-mediated drug-resistance [18, 19]. miR-1291 has been demonstrated to affect drug disposition and increase chemosensitivity by targeting MRP1 in PANC-1 cells [19]. miR-133a and miR-326 have also been reported to induce drug accumulation by suppressing MRP1 expression in HepG2 cells [18]. In the present study, we transfected miR-145 mimics and MRP1 siRNA, respectively, into MCF-7 cells, MRP1 expression level decreased (Supplementary Figure S4 and Figure 4G), conversely, transfecting miR-145 inhibitor into MCF-7 cells increased MRP1 expression (Figure 4J), these data suggested MRP1 was the target of miR-145 and miR-145 negatively regulated MRP1 expression. In luciferase assay, we constructed a series plasmids which contained human MRP1 3′UTR region fragment, co-transfecting miR-145 and wild type plasmid decreased luciferase activity, and then we mutated the binding site, co-transfecting miR-145 and mutant plasmid reversed luciferase activity compared with co-transfecting miR-145 and wild type plasmid (Figure 3B), these results suggested MRP1 was the direct target of miR-145. Further study, we proved miR-145 sensitized breast cancer cells to doxorubicin via inducing intracellular doxorubicin accumulation by inhibiting MRP1, synthetic or stably expressed miR-145 reversed resistance to doxorubicin *in vitro* and *in vivo* by targeting MRP1, miR-145 exerts the potential to be developed for MDR inhibitors (Figure 4 and 5).

Here, our results showed MRP1 expression was markedly increased in doxorubicin resistant MCF-7/ADR cells compared to parental MCF-7 cells. miR-145 overexpression increased intracellular doxorubicin accumulation in MCF-7 cells (Figure 2C), accompanied by increased sensitivity to doxorubicin (Figure 2B). And, this observation could also be seen in doxorubicin resistant MCF-7/ADR cells (Figure 2E and 2F), suggesting that miR-145 sensitized both MCF-7 and MCF-7/ADR to doxorubicin. However, miR-145 overexpression decreased the IC_50_ of doxorubicin in MCF-7ADR cells from 74 to 20uM, which was still higher than the IC_50_ of MCF-7 (2uM) in MCF-7 cells. Moreover, suppression of miR-145 increased the IC_50_ of doxorubicin in MCF-7 cells from 0.9 to 3uM, which was still lower than the IC_50_ of doxorubicin in MCF-7/ADR cells. These data proved that miR-145 overexpression partially reversed resistance to doxorubicin in MCF-7/ADR cells, and suppression of miR-145 partially got MCF-7 cells resistant to doxorubicin. These findings indicated that other mechanisms except for MRP1-mediated drug resistance may be associated in breast cancer cells to doxorubicin. Serguienko et al. demonstrated that let-7a increased cells sensitivity to doxorubicin by regulating energy metabolism [28]. Jiang and his colleagues proved that forced-expression of miR-489 reversed resistance to doxorubicin in doxorubicin resistant MCF-7/ADM cells by regulating epithelial-mesenchymal transition (EMT) properties [29]. And, there is report showed that ectopic miR-34a expression increases sensitivity to doxorubicin by directly targeting NOTCH1 and decreasing cancer stem cell properties [30].

Our results revealed that miR-145 expression level decreased significantly both in breast cancer tissues and breast cancer cell lines compared the control. In addition, our data showed that miR-145 expression level was reduced in doxorubicin resistant MCF-7/ADR versus MCF-7 cells (Figure 1G). And miR-145 expression level increased at the beginning then decreased markedly and maintained in low level in the process of inducing doxorubicin resistance in MCF-7 cells (Figure 1F). The underling mechanisms of the alterations of miR-145 expression level are still unclear. According to the previous study, doxorubicin was p53 inducer and p53 transcriptionally induced miR-145 expression by binding to the p53 response element in the upstream of miR-145 promoter region. So, miR-145 level may be induced via p53 by doxorubicin at the beginning. And, DNA hypermethylation and p53 mutation suppressed miR-145 expression by affecting p53 binding to the p53 response element [31, 32]. DNA methylation and p53 mutation accounted at least part for the suppression of miR-145 in cancers, which may be also one of the mechanisms for the reduction of miR-145 in doxorubicin resistant MCF-7/ADR cells.

In conclusion, our data revealed higher MRP1 expression and lower miR-145 expression in breast cancer. Further, MRP1 was negatively regulated at the posttranscriptional level by miR-145 through a specific target motif at nt 1728-1734 of the MRP1 3′UTR. Moreover, miR-145 sensitized breast cancer to ADR chemotherapy *in vitro* and *in vivo* by reducing the MRP1 expression level and increasing the intracellular concentration of ADR.

miRBase Sequence Database [33]; the TCGA database [34]; TargetScan [35]; and MicroCosm [36] were used in this manuscript as bioinformatics tools.

## MATERIALS AND METHODS

### Clinical cancer samples and cell lines

All cancer samples were obtained from the Wuxi Maternity and Children Health Hospital (Wuxi, China) and were stored in liquid nitrogen until analysis. All experiments were conducted in accordance with the

Declaration of Helsinki and were approved by the Wuxi Maternity and Children Health Hospital Ethics Committee in the hospital.

The breast cancer cell lines MCF-7, MDA-MB-231, MDA-MB-453, MDA-MB-468, MCF-10A, MDA-kb2, and MCF-7/ADR were used in this study. The MCF-7, MDA-MB-453, and MDA-MB-468 cells were obtained from the cell bank of Shanghai Institute for Biological Sciences. The MCF-10A, MDA-kb2 cells and MCF-7/ADR cells were obtained from FuDan Cell Center (Shanghai, China). MCF-7 cells were cultured in complete high-glucose DMEM (Hyclone, Logan, UT, USA) supplemented with 10% FBS (Gibco, Carlsbad, CA, USA) and 1% antibiotic-antimycotic (Gibco, Carlsbad, CA, USA). MDA-MB-231, MDA-MB-453, MDA-MB-468 and MDA-kb2 cells were cultured in Leibovitz's L-15 Medium containing 10% FBS. MCF-10A cells were cultured in DMEM/F12 medium containing 5% horse serum, 1% penicillin/streptomycin, 20 ng/ml EGF, 0.5 μg/ml hydrocortisone, 100 ng/ml Cholera toxin and 10 μg/ml insulin. MCF-7/ADR cells were cultured in RPMI 1640 medium (Gibco, Carlsbad, CA, USA) supplemented with 10% FBS (Gibco, Carlsbad, CA, USA) and 1% antibiotic-antimycotic (Gibco, Carlsbad, CA, USA). MCF-7, MCF-7/ADR and MCF-10A cells were cultured at 37°C in 5% CO_2_, and all other cell types were cultured in 100% air.

### Reverse transcription (RT)-polymerase chain reaction (PCR) and real-time PCR

Total RNA was isolated using a UNIQ-10/Trizol total RNA extraction kit (Sangon, Shanghai, China) and was reverse-transcribed into cDNA using the PrimeScript RT Reagent Kit (Takara, Otus, Shiga, Japan). The primer sets used are listed in Supplementary Table 1. Quantitative real-time RT-PCR (qRT-PCR) analysis was performed using SYBR Premix Ex Taq (Takara).

miRNAs were isolated using the mirVana miRNA Isolation Kit (Ambion, Austin, TX) according to the manufacturer's instructions. RT and miRNA detection were conducted using the NCode VILO miRNA cDNA Synthesis Kit and the EXPRESS SYBR GreenER miRNA qRT-PCR Kit, respectively (Invitrogen, Carlsbad, CA, USA). miRNA-specific forward primers were designed according to the manufacturer's instructions; these primers are also listed in Supplementary Table 1. The observed miRNA expression levels were normalized to the U6 expression levels.

### Construction of luciferase reporter plasmids

The 3′UTR of human ABCC1 (NM_004996, 1792 bp, GenBank) was amplified via PCR using the genomic DNA of MCF-7. The primers used are listed in Supplementary Table 1. This PCR fragment was cloned into the psiCHECK-2 vector (Promega, Madison, WI, USA) using the In-fusion Advantage PCR Cloning Kit (Clontech, Mountain View, CA, USA).

### Site-directed mutant luciferase reporter plasmids

The mutated plasmid was cloned using the KOD-Plus-Mutagenesis Kit (Toyobo, Osaka, Japan) using standard primers (Supplementary Table 1). DNA sequencing confirmed the nucleotide sequence of these plasmids.

### Luciferase assays

For luciferase reporter assays, various luciferase reporter plasmids were co-transfected with 50 nM miRNA mimics or their seed region mutants (GenePharma, Shanghai, China) into MCF-7 cells using Lipofectamine 2000 (Invitrogen). Luciferase activity was analyzed after 72 hours using the Dual-Luciferase Reporter Assay System according to the manufacturer's protocol (Promega).

### Western blotting

Total protein samples were lysed in RIPA buffer (150 mM NaCl, 0.1% SDS, 0.5% sodium deoxycholate, 1% NP-40 and 50 mM Tris-HCl, pH 7.6) containing a protein inhibitor cocktail (Roche, Mannheim, Germany). After separation on 8% polyacrylamide gels and transfer to a 0.45 μm membrane (Millipore, Billerica, MA, USA), the proteins were detected using anti-MRP1 (Abcam, Hong Kong, China) and anti-GAPDH (Sigma) antibodies.

### 3-(4,5-dimethyl-2-thiazolyl)-2,5-diphenyl-2H-tetrazolium bromide (MTT) assays

Cells were seeded on 96-well plates (6 × 10^3^ cells per well) and incubated for 24 h in 100 μl of medium. Then, the cells were treated with various concentrations of doxorubicin. After incubation for 48 h, 20 μl of 5 mg/ml MTT (Sigma) dissolved in phosphate-buffered saline (PBS) was added to the cells. After reaction for 4 h at 37°C, 100 μl of dimethyl sulfoxide (DMSO) was added. The absorbance at 570 nm was measured using a BioTek SYNFRGY4 microplate reader (BioTek, Winooski, VT, USA).

### Intracellular ADR concentration analysis

Cells were seeded on 6-well plates (2× 10^5^ cells per well) and cultured overnight. Then, the cells were transfected with 50 nM miR-145 mimic/inhibitor, or ABCC1 siRNA (GenePharma) or 50 nM negative control (NC) siRNA using Lipofectamine 2000 (Invitrogen) according to the manufacturer's instructions. After 48 h, the MCF-7 or MCF-7/ADR cells were incubated in medium containing ADR (50 μM or 5 mM, respectively, Merck) for 1 h. Then, the cells were washed twice with ice-cold PBS, harvested via centrifugation, and subjected to flow cytometry using a FACS Calibur flow cytometer (BD Biosciences, Franklin Lakes, NJ, USA) in the FL-3 channel (650-630 nm).

### *In vivo* drug sensitivity assay

BALB/c athymic nude mice (female, 4–6 weeks old and 16–20 g) were bred under pathogen-free conditions in the Animal Center of Shanghai Institute of Materia Medica. All animal experiments were performed in accordance with the National Institutes of Health Guide for the Care and Use of Laboratory Animals. Stable transfectants overexpressing miR-145 were generated via lentiviral transduction using a GV369-GFP vector (GeneChem Co., Ltd., Shanghai, China). As a control, we generated a lentiviral vector (LV) that expressed green fluorescent protein alone (LV-GFP). Approximately 5×10^6^ MDA-MB-231 cells stably transfected with lenti-miR-145 or the control vector were subcutaneously injected into the right fat pad of nude mice. Two weeks later, ADR (2.5 mg/kg) was administered intravenously once a week for 4 weeks. Tumor growth was monitored via measurement using a caliper every three days until termination of the experiment. The tumor volume (V) was determined based on the length (L) and the width (W) according to the following formula: V = (L×W^2^)×0.5. The mice were humanely sacrificed after 28 days, and the tumors were weighed and photographed. Western blotting and qRT-PCR were performed to determine the MRP1 and miR-145 expression levels and the concentration of ADR.

### Statistical analysis

Data was analyzed by Student's test unless otherwise mentioned. Values are expressed as the mean ±SEM. All reported p values were two-sided. *p* values for the tumor volume groups measurement were determined by two way ANOVA. p<0.05 was considered statistically significant, and we note that, throughout this paper, *p<0.05, **or ## *p*<0.01,*** or ### P< 0.001, NS is not significant.

## SUPPLEMENTARY FIGURES AND TABLE



## Abbreviations

ADR: Adriamycin (Doxorubicin)
DMEM: Dulbecco's modified Eagle's medium
FBS: fetal bovine serum
miRNA: microRNA
MTT: 3-(4, 5-dimethyl-2-thiazolyl)-2, 5-diphenyl-2H-tetrazolium bromide
PBS: phosphate-buffered saline
UTR: untranslated region
TNBC: Triple Negative Breast Cancer
ABC: ATP-binding cassette
