# Supplementary material for: miR-145 sensitizes breast cancer to doxorubicin by targeting multidrug resistance-associated protein-1
Source: Oncotarget. 2016 Jul 26;7(37):59714–26. doi: 10.18632/oncotarget.10845 (PMC5312343; doi:10.18632/oncotarget.10845)
Supplement: Supplementary file 1 [file oncotarget-07-59714-s001.pdf]

miR-145 sensitizes breast cancer to doxorubicin by targeting multidrug resistance-associated protein-1

SUPPLEMENTARY FIGURES AND TABLE

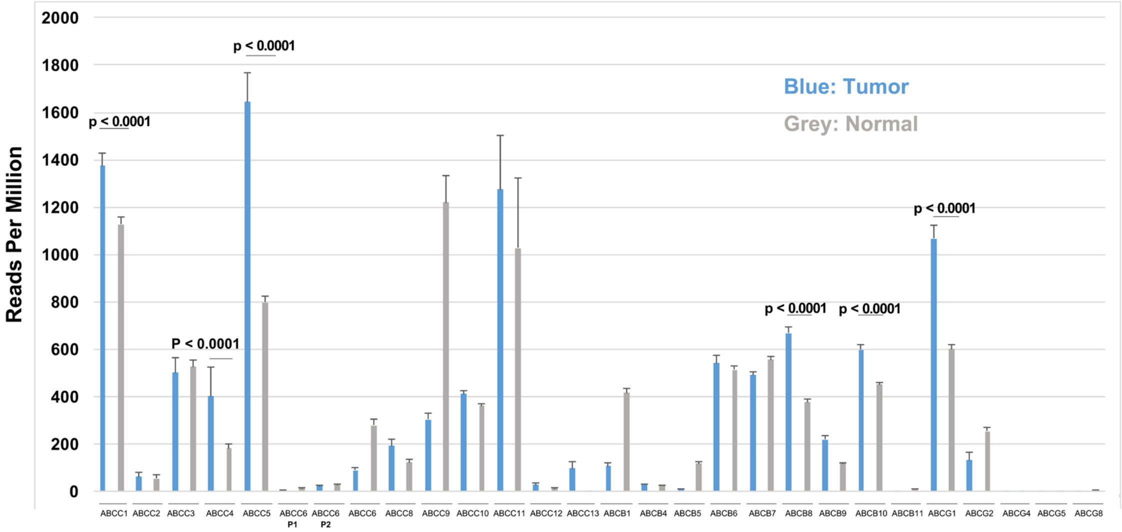

Supplementary Figure S1: MiRNAs expression level in breast cancer (and the matched normal breast tissues).

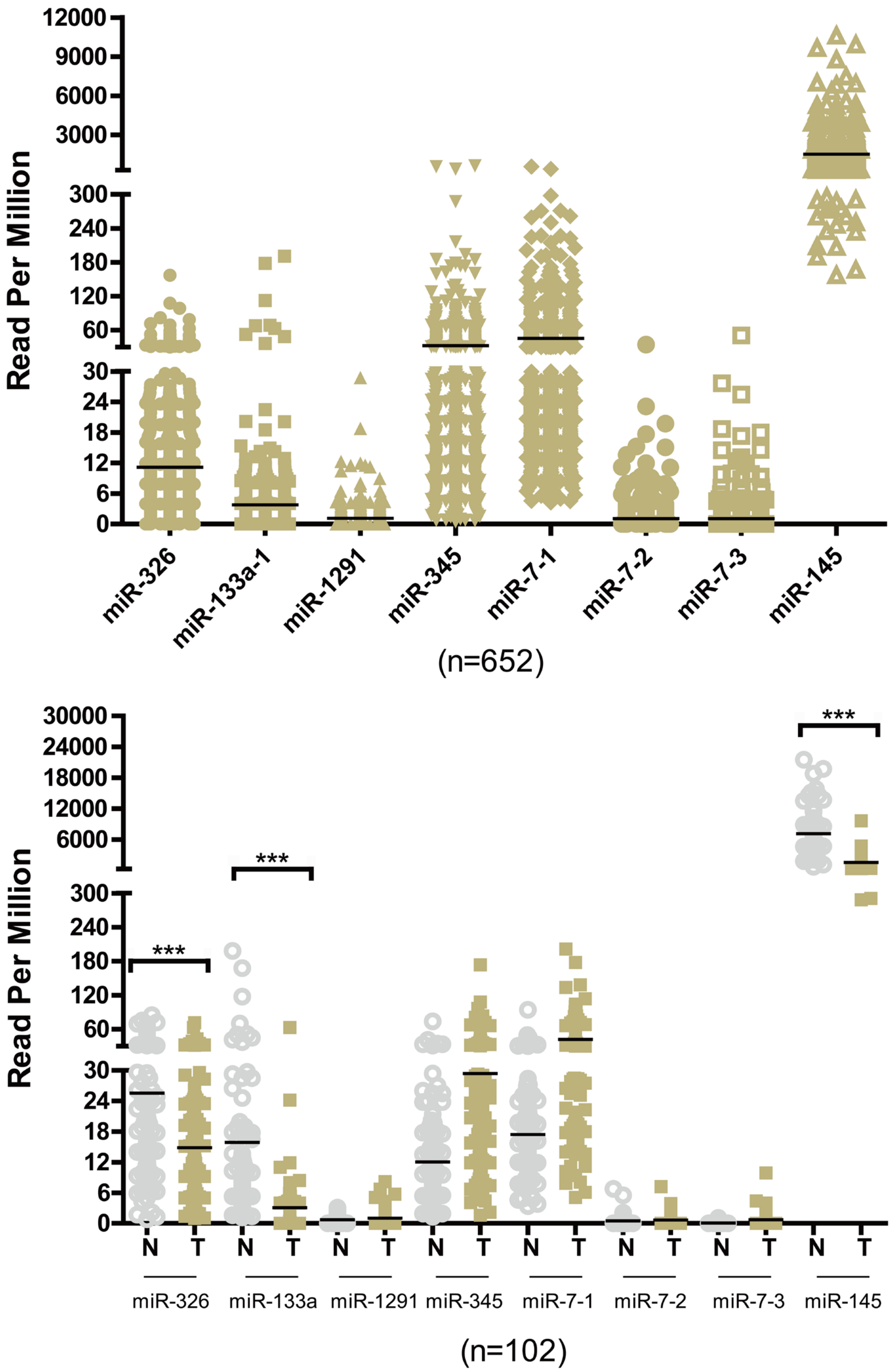

Supplementary Figure S2: Pearson correlation analysis of miR-145 and MRP1 expression level in breast cancer cell lines.

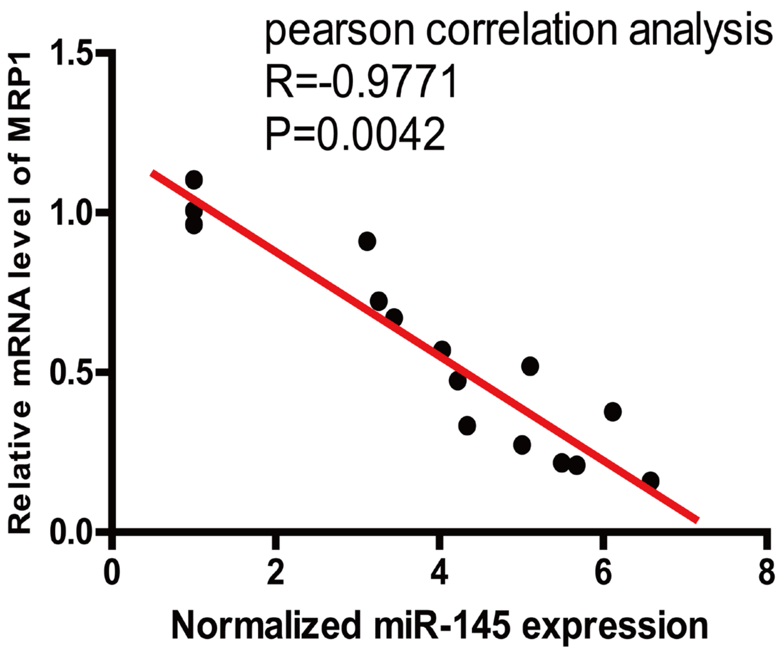

Supplementary Figure S3: ABC transporters mRNA expression level in breast cancer.

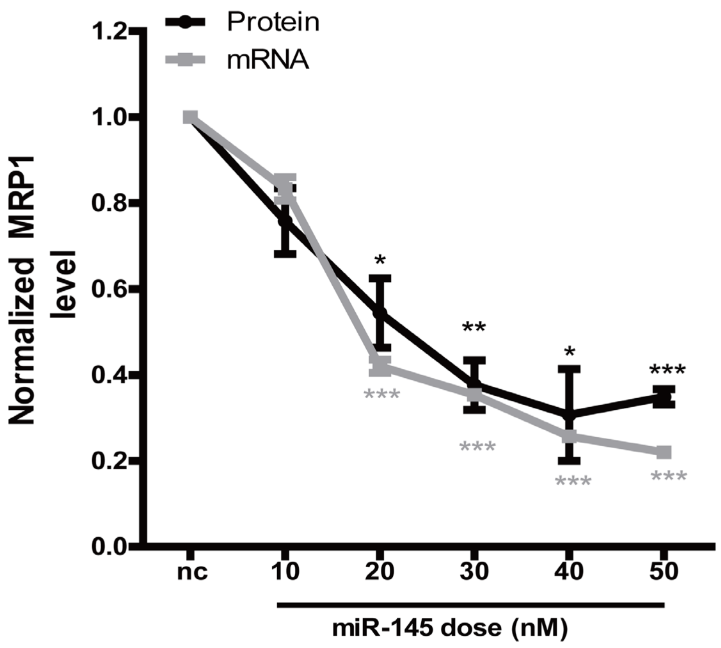

Supplementary Figure S4: MRP1 mRNA and protein expression level in MCF-7 cells transfected with indicated dose miR-145 mimics.

Supplementary Table S1: Oligonucleotides used for the construction of plasmids or for quantitative polymerase chain reaction

Application	Oligonucleotide	Sequence (5’→3’)
Cloning		
psiCHECK-ABCC1-3’UTR	Forward	CCTCGAGAAAGACGCCGGCTTGGTGTG
	Reverse	TTGCGGCCGCCTGCAAATGTTCGCATTTCAGTGC
psiCHECK2-ABCC1-3’UTR(mutant)	Forward	TGACTTACTGACCTTATGCGAACGTTTGCAGGCGG
	Reverse	GTTCGCATAAGGTCAGTAAGTCATTACAAAAGGAT
Universal reverse primer		
qPCR		
MRP1	Forward	GTCGGGGCATATTCCTGGC
	Reverse	GGGCATCCTCTTTTAAGGCTG
GAPDH	Forward	GGTGGTCTCCTCTGACTTCAACA
	Reverse	GTTGCTGTAGCCAAATTCGTTGT
miR-145	Forward	TCCAGTTTTCCCAGGAATCCCT
U6	Forward	GCTTCGGCAGCACATATACTAAAAT
	Reverse	CGCTTCACGAATTTGCGTGTCAT
